# Plasma galectin-3 concentration and estimated glomerular filtration rate in patients with type 2 diabetes with and without albuminuria

**DOI:** 10.1038/s41598-022-20860-x

**Published:** 2022-09-29

**Authors:** Jin Ook Chung, Seon-Young Park, Seung Baek Lee, Na-Ri Kang, Dong Hyeok Cho, Dong Jin Chung, Min Young Chung

**Affiliations:** 1 Division of Endocrinology and Metabolism, Department of Internal Medicine, Chonnam National University Medical School, 8 Hak-Dong, Dong-Gu, Gwangju, 501-757 Republic of Korea; 2 Division of Gastroenterology and Hepatology, Department of Internal Medicine, Chonnam National University Medical School, 8 Hak-Dong, Dong-Gu, Gwangju, 501-757 Republic of Korea; 3 Division of Radiology, Mayo Clinic, Rochester, MN 55905 USA; 4 Department of Molecular Pharmacology and Experimental Therapeutics, Mayo Clinic, Rochester, MN 55905 USA

**Keywords:** Endocrinology, Endocrine system and metabolic diseases

## Abstract

This study aimed to investigate the association between galectin-3 concentration and estimated glomerular filtration rate (eGFR) in patients with type 2 diabetes mellitus (T2DM) with and without albuminuria. In this cross-sectional study, we examined 334 patients with T2DM. The eGFR was calculated using a creatinine-based formula (eGFR_crea_) and a combined creatinine-cystatin C equation (eGFR_crea-cyst_). The participants were categorized into two groups based on the urinary albumin-to-creatinine ratio (UACR): patients without albuminuria (UACR < 30 mg/g) and those with albuminuria (UACR ≥ 30 mg/g). Greater concentrations of plasma galectin-3 were associated with lower eGFR_crea-cyst_ and eGFR_crea_ levels in patients with and without albuminuria. Plasma galectin-3 concentrations were negatively correlated with eGFR_crea-cyst_ in patients with normoalbuminuria and albuminuria (γ = − 0.405, *P* < 0.001; γ = − 0.525, *P* < 0.001, respectively). Galectin-3 concentrations were significantly associated with eGFR_crea-cyst_ after adjusting for sex, age, and other confounding factors, including UACR as a categorical or continuous variable in multiple regression analyses (β = − 0.294, 95% CI − 70.804 to − 41.768, *P* < 0.001; β = − 0.265, 95% CI − 65.192 to − 36.550, *P* < 0.001, respectively). Likewise, when eGFR_crea-cyst_ was treated in place of eGFR_crea_, this result was replicated in the correlation and regression analyses. Galectin-3 concentration was negatively associated with eGFR in patients with T2DM, independent of albuminuria status.

## Introduction

Type 2 diabetes mellitus (T2DM) is a chronic metabolic disease that is characterized by chronic hyperglycemia^1,2^. The prevalence of T2DM is increasing, with a substantial burden on clinical and public health owing to its complications^1^. Chronic hyperglycemia plays a pivotal role in progressive end-organ damage in patients with T2DM^2^. Diabetic nephropathy is a common microvascular complication and an important cause of end-stage kidney disease, which may require dialysis or kidney transplantation^2^. A large body of evidence demonstrates that albuminuria is an important risk factor for progressive kidney failure and cardiovascular disease in patients with T2DM^3–6^. In addition, reduced glomerular filtration rate (GFR) has been suggested to be independently linked to the risk of detrimental cardiovascular and kidney outcomes in patients with T2DM^3,7^.

According to the traditional view, the natural history of diabetic nephropathy is characterized by a progressive increase in urinary albumin excretion from normoalbuminuria to microalbuminuria and, subsequently, to macroalbuminuria^8,9^. Furthermore, progression from microalbuminuria to macroalbuminuria is assumed to initiate renal function loss^9^. However, recent evidence suggests that an initial decline in GFR can occur in the setting of normal urinary albumin excretion^10^, indicating that albuminuria may not be a sensitive marker for early diabetic nephropathy^11^. Autopsy studies of patients with diabetes suggest that diabetes-related histopathological lesions in the kidney occur before the onset of albuminuria^11–13^. Therefore, in a clinical setting, it is crucial to identify the factors with implications for early kidney damage in patients with diabetes.

Galectin‐3, which belongs to the galectin family, is a β-galactoside‐binding protein mainly composed of a C-terminal carbohydrate-recognition domain and an N-terminal domain^14^. Galectin-3 is ubiquitously expressed in various cells and tissues and secreted into the bloodstream^14^. Its structural property allows galectin-3 to bind several proteins, thus exerting multiple context-dependent biological functions^14,15^. Recently, galectin-3 has been implicated in inflammation and fibrosis^14,15^. In addition, galectin-3 is linked to tissue injury at an early stage^16^. In a preclinical study, galectin-3 was found to be associated with renal injury^17^. Galectin-3 expression was rapidly up-regulated in models of renal damage^18^. In community-based studies, elevated concentrations of plasma galectin-3 were associated with an increased risk of chronic kidney disease^19^. In a renal biopsy-based study, plasma galectin-3 concentrations were positively correlated with renal fibrosis and inversely correlated with eGFR^20^. In addition, a longitudinal study of patients with T2DM showed that high concentrations of galectin-3 were associated with a doubling of serum creatinine levels and incident macroalbuminuria. However, little is known about the contribution of galectin-3 to early renal function decline in patients with T2DM and normoalbuminuria.

Therefore, this study aimed to evaluate the relationship between galectin-3 concentration and eGFR in patients with T2DM with and without albuminuria.

## Results

Table 1 summarizes the characteristics of the patients with T2DM in this study. Patients with albuminuria had higher systolic blood pressure, a longer duration of diabetes, higher A1C levels, higher triglyceride levels, and a higher prevalence of use of insulin and angiotensin converting enzyme inhibitors (ACEi)/angiotensin II receptor blockers (ARB). The eGFR_crea-cyst_ and eGFR_crea_ were significantly lower in patients with albuminuria than in those with normoalbuminuria. In addition, plasma galectin-3 concentrations were significantly higher in patients with albuminuria than those in patients with normoalbuminuria.

**Table 1:** Characteristics of patients with T2DM with and without albuminuria.

	Albuminuria (−) (n = 217)	Albuminuria (+) (n = 117)	*P-*value*
Age (years; mean [SD])	58.4 (12.6)	59.5 (13.2)	0.455
Smoking, n (%)	27 (12.4)	19 (16.2)	0.337
Men, n (%)	108 (49.8)	71 (60.7)	0.056
BMI (kg/m^2^; mean [SD])	25.6 (4.4)	25.6 (3.9)	0.914
Hyperlipidemia, n (%)	157 (72.4)	89 (76.1)	0.462
Hypertension, n (%)	126 (58.1)	75 (64.1)	0.282
Systolic BP (mmHg; mean [SD])	131.7 (16.8)	138.7 (20.1)	0.001
Diastolic BP (mmHg; mean [SD])	76.1 (11.4)	77.3 (11.4)	0.366
Diabetes duration (years; median [25th, 75th])	3.5 (0.5, 10.0)	12.0 (3.0, 20.0)	< 0.001
A1C (%; mean [SD])	7.3 (1.5)	8.5 (2.0)	< 0.001
A1C (mmol/mol; mean [SD])	57 (17)	69 (22)	< 0.001
Hgb (g/L; mean [SD])	141.3 (15.8)	139.1 (20.6)	0.326
Triglyceride (mmol/L; median [25th, 75th])	1.3 (0.9, 2.0)	1.6 (1.1, 2.3)	0.002
Total cholesterol (mmol/L; median [25th, 75th])	4.1 (3.4, 5.2)	4.3 (3.5, 5.4)	0.654
LDL-C (mmol/L; median [25th, 75th])	2.4 (1.9, 3.0)	2.5 (1.9, 3.1)	0.706
HDL-C (mmol/L; median [25th, 75th])	1.3 (1.1, 1.5)	1.3 (1.0, 1.4)	0.323
Galectin-3 (ng/mL; median [25th, 75th])	8.54 (6.87, 10.35)	9.41 (7.71, 11.03)	0.007
UACR (mg/g; median [25th, 75th])	9.2 (5.7, 14.1)	126.3 (56.2, 461.0)	< 0.001
eGFR_crea-cyst_ (mL/min/1.73m^2^; mean [SD])	94.2 (20.9)	76.9 (32.1)	< 0.001
Classification, n (%)			
≥ 90	138 (63.6)	42 (35.9)	< 0.001
60–89	62 (28.6)	39 (33.3)	
< 60	17 (7.8)	36 (30.8)	
eGFR_crea_ (mL/min/1.73m^2^; mean [SD])	94.1 (17.4)	79.0 (28.1)	< 0.001
Classification, n (%)			
≥ 90	150 (69.1)	51 (43.6)	< 0.001
60–89	54 (24.9)	36 (30.8)	
< 60	13 (6.0)	30 (25.6)	
Use of insulin, n (%)	29 (13.4)	41 (35.0)	< 0.001
Use of OHAs, n (%)	158 (72.8)	90 (76.9)	0.412
ACEi/ARB, n (%)	74 (34.1)	69 (59.0)	< 0.001
SGLT2i, n (%)	11 (7.1)	12 (13.0)	0.124
GLP1-RA, n (%)	1 (0.6)	1 (0.6)	‒
Statin, n (%)	140 (64.5)	76 (65.0)	0.936

Data presented as mean (SD) and median (25th, 75th percentile).

*A1C* glycated hemoglobin, *ACEi* angiotensin converting enzyme inhibitor, *ARB* angiotensin II receptor blocker, *BMI* body mass index, *BP* blood pressure, *eGFR* estimated glomerular filtration rate, *GLP1-RA* glucagon-like peptide-1 receptor agonist, *Hgb* hemoglobin, *HDL-C* high-density lipoprotein cholesterol, *LDL-C* low-density lipoprotein cholesterol, *OHA* oral hypoglycemic agent, *SD* standard deviation, *SGLT2i* sodium-glucose cotransporter 2 inhibitor, *T2DM* type 2 diabetes mellitus, *UACR* urinary albumin-to-creatinine ratio.

*Student's t-test and the Mann–Whitney U test for parametric and nonparametric continuous variables (diabetes duration, triglyceride, total cholesterol, LDL-C, HDL-C, Galectin-3, and UACR); the chi-squared test for categorical variables.

We subdivided patients with T2DM into with and without albuminuria at an overall median galectin-3 level of 8.73 ng/mL (Tables 2, 3). In both groups, higher concentrations of galectin-3 were associated with older age, longer duration of diabetes, and higher UACR (Tables 2, 3). In addition, higher concentrations of galectin-3 were associated with lower eGFR_crea-cyst_ and eGFR_crea_.

**Table 2:** Characteristics of patients with T2DM without albuminuria.

	Albuminuria (−)		*P-*value*
	Galectin-3 < 8.73 (n = 118)	Galectin-3 ≥ 8.73 (n = 99)	
Galectin-3 (ng/mL; median [25th, 75th])	6.95 (5.96, 7.81)	10.49 (9.62, 12.57)	< 0.001
Age (years; mean [SD])	55.2 (12.3)	62.2 (11.8)	< 0.001
Smoking, n (%)	15 (12.7)	12 (12.1)	0.896
Men, n (%)	60 (50.8)	48 (48.5)	0.729
BMI (kg/m^2^; mean [SD])	25.5 (4.9)	25.7 (3.7)	0.826
Hyperlipidemia, n (%)	79 (66.9)	78 (78.8)	0.052
Hypertension, n (%)	59 (50.0)	67 (67.7)	0.009
Systolic BP (mmHg; mean [SD])	130.6 (15.5)	133.0 (18.2)	0.300
Diastolic BP (mmHg; mean [SD])	75.9 (9.9)	76.5 (13.0)	0.688
Diabetes duration (years; median [25th, 75th])	3.0 (0.2, 9.0)	5.0 (1.0, 12.0)	0.012
A1C (%; mean [SD])	7.2 (1.5)	7.5 (1.6)	0.164
A1C (mmol/mol; mean [SD])	55 (16)	58 (17)	0.164
Hgb (g/L; mean [SD])	141.1 (15.0)	141.5 (16.7)	0.872
Triglyceride (mmol/L; median [25th, 75th])	1.3 (0.9, 2.0)	1.3 (1.0, 1.9)	0.257
Total cholesterol (mmol/L; median [25th, 75th])	4.6 (3.8, 5.4)	3.8 (3.3, 4.8)	< 0.001
LDL-C (mmol/L; median [25th, 75th])	2.6 (2.1, 3.2)	2.2 (1.8, 2.9)	0.002
HDL-C (mmol/L; median [25th, 75th])	1.3 (1.1, 1.5)	1.3 (1.1, 1.4)	0.080
UACR (mg/g; median [25th, 75th])	8.4 (5.3, 11.6)	10.1 (6.7, 16.1)	0.016
eGFR_crea-cyst_ (mL/min/1.73m^2^; mean [SD])	102.1 (16.1)	84.9 (22.0)	< 0.001
Classification, n (%)			
≥ 90	92 (78.0)	46 (46.5)	< 0.001
60–89	23 (19.5)	39 (39.4)	
< 60	3 (2.5)	14 (14.1)	
eGFR_crea_ (mL/min/1.73m^2^; mean [SD])	99.5 (13.5)	87.8 (19.3)	< 0.001
Classification, n (%)			
≥ 90	100 (84.7)	50 (50.5)	< 0.001
60–89	16 (13.6)	38 (38.4)	
< 60	2 (1.7)	11 (11.1)	
Use of insulin, n (%)	11 (9.3)	18 (18.2)	0.056
Use of OHAs, n (%)	82 (69.5)	76 (76.8)	0.230
ACEi/ARB, n (%)	35 (29.7)	39 (39.4)	0.132
SGLT2i, n (%)	2 (2.5)	9 (12.3)	0.018
GLP1-RA, n (%)	1 (1.2)	0 (0.0)	‒
Statin, n (%)	71 (60.2)	69 (69.7)	0.144

Median galectin-3 for all patients (8.73 ng/mL) was unequally divided into normoalbuminuric and albuminuric groups. Data presented as mean (SD) and median (25th, 75th percentile).

*A1C* glycated hemoglobin, *ACEi* angiotensin converting enzyme inhibitor, *ARB* angiotensin II receptor blocker, *BMI* body mass index, *BP* blood pressure, *eGFR* estimated glomerular filtration rate, *GLP1-RA* glucagon-like peptide-1 receptor agonist, *Hgb* hemoglobin, *HDL-C* high-density lipoprotein cholesterol, *LDL-C* low-density lipoprotein cholesterol, *OHA* oral hypoglycemic agent, *SD* standard deviation, *SGLT2i* sodium-glucose cotransporter 2 inhibitor, *T2DM* type 2 diabetes mellitus, *UACR* urinary albumin-to-creatinine ratio.

*Student's t-test and the Mann–Whitney U test for parametric and nonparametric continuous variables (diabetes duration, triglyceride, total cholesterol, LDL-C, HDL-C, Galectin-3, and UACR); the chi-squared test for categorical variables.

**Table 3:** Characteristics of patients with T2DM with albuminuria.

	Albuminuria (+)		*P-*value*
	Galectin-3 < 8.73 (n = 49)	Galectin-3 ≥ 8.73 (n = 68)	
Galectin-3 (ng/mL; median [25th, 75th])	7.52 (6.47, 8.18)	10.72 (9.66, 12.52)	< 0.001
Age (years; mean [SD])	55.9 (13.5)	62.1 (12.4)	0.011
Smoking, n (%)	5 (10.2)	14 (20.6)	0.133
Men, n (%)	33 (67.3)	38 (55.9)	0.210
BMI (kg/m^2^; mean [SD])	26.4 (4.0)	25.1 (3.7)	0.081
Hyperlipidemia, n (%)	34 (69.4)	55 (80.9)	0.151
Hypertension, n (%)	28 (57.1)	47 (69.1)	0.183
Systolic BP (mmHg; mean [SD])	136.8 (17.6)	140.0 (21.8)	0.386
Diastolic BP (mmHg; mean [SD])	79.2 (10.3)	76.0 (11.1)	0.138
Diabetes duration (years; median [25th, 75th])	10.0 (1.8, 16.5)	13.5 (6.0, 20.0)	0.026
A1C (%; mean [SD])	8.6 (2.0)	8.4 (2.0)	0.705
A1C (mmol/mol; mean [SD])	70 (22)	69 (22)	0.705
Hgb (g/L; mean [SD])	144.8 (14.3)	135.1 (23.4)	0.007
Triglyceride (mmol/L; median [25th, 75th])	1.6 (1.1, 2.2)	1.6 (1.1, 2.4)	0.631
Total cholesterol (mmol/L; median [25th, 75th])	4.5 (3.7, 5.5)	4.0 (3.4, 5.2)	0.221
LDL-C (mmol/L; median [25th, 75th])	2.6 (1.9, 3.2)	2.4 (1.9, 3.1)	0.225
HDL-C (mmol/L; median [25th, 75th])	1.3 (1.1, 1.6)	1.2 (1.0, 1.4)	0.061
UACR (mg/g; median [25th, 75th])	91.8 (42.9, 205.2)	201.4 (71.0, 794.0)	0.001
eGFR_crea-cyst_ (mL/min/1.73m^2^; mean [SD])	92.5 (28.7)	65.6 (29.8)	< 0.001
Classification, n (%)			
≥ 90	26 (53.1)	16 (23.5)	< 0.001
60–89	16 (32.7)	23 (33.8)	
< 60	7 (14.3)	29 (42.6)	
eGFR_crea_ (mL/min/1.73m^2^; mean [SD])	91.3 (24.0)	70.1 (27.7)	< 0.001
Classification, n (%)			
≥ 90	31 (63.3)	20 (29.4)	< 0.001
60–89	14 (28.6)	22 (32.4)	
< 60	4 (8.2)	26 (38.2)	
Use of insulin, n (%)	15 (30.6)	26 (38.2)	0.394
Use of OHAs, n (%)	35 (71.4)	55 (80.9)	0.231
ACEi/ARB, n (%)	24 (49.0)	45 (66.2)	0.062
SGLT2i, n (%)	3 (8.3)	9 (16.1)	0.354
GLP1-RA, n (%)	0 (0.0)	1 (1.8)	‒
Statin, n (%)	30 (61.2)	46 (67.6)	0.473

Median galectin-3 for all patients (8.73 ng/mL) was unequally divided into normoalbuminuric and albuminuric groups. Data presented as mean (SD) and median (25th, 75th percentile).

*A1C* glycated hemoglobin, *ACEi* angiotensin converting enzyme inhibitor, *ARB* angiotensin II receptor blocker, *BMI* body mass index, *BP* blood pressure, *eGFR* estimated glomerular filtration rate, *GLP1-RA* glucagon-like peptide-1 receptor agonist, *Hgb* hemoglobin, *HDL-C* high-density lipoprotein cholesterol, *LDL-C* low-density lipoprotein cholesterol, *OHA* oral hypoglycemic agent, *SD* standard deviation, *SGLT2i* sodium-glucose cotransporter 2 inhibitor, *T2DM* type 2 diabetes mellitus, *UACR* urinary albumin-to-creatinine ratio.

*Student's t-test and the Mann–Whitney U test for parametric and nonparametric continuous variables (diabetes duration, triglyceride, total cholesterol, LDL-C, HDL-C, Galectin-3, and UACR); the chi-squared test for categorical variables; Fisher’s exact test for a categorical variable (SGLT2i).

**Figure 1:**
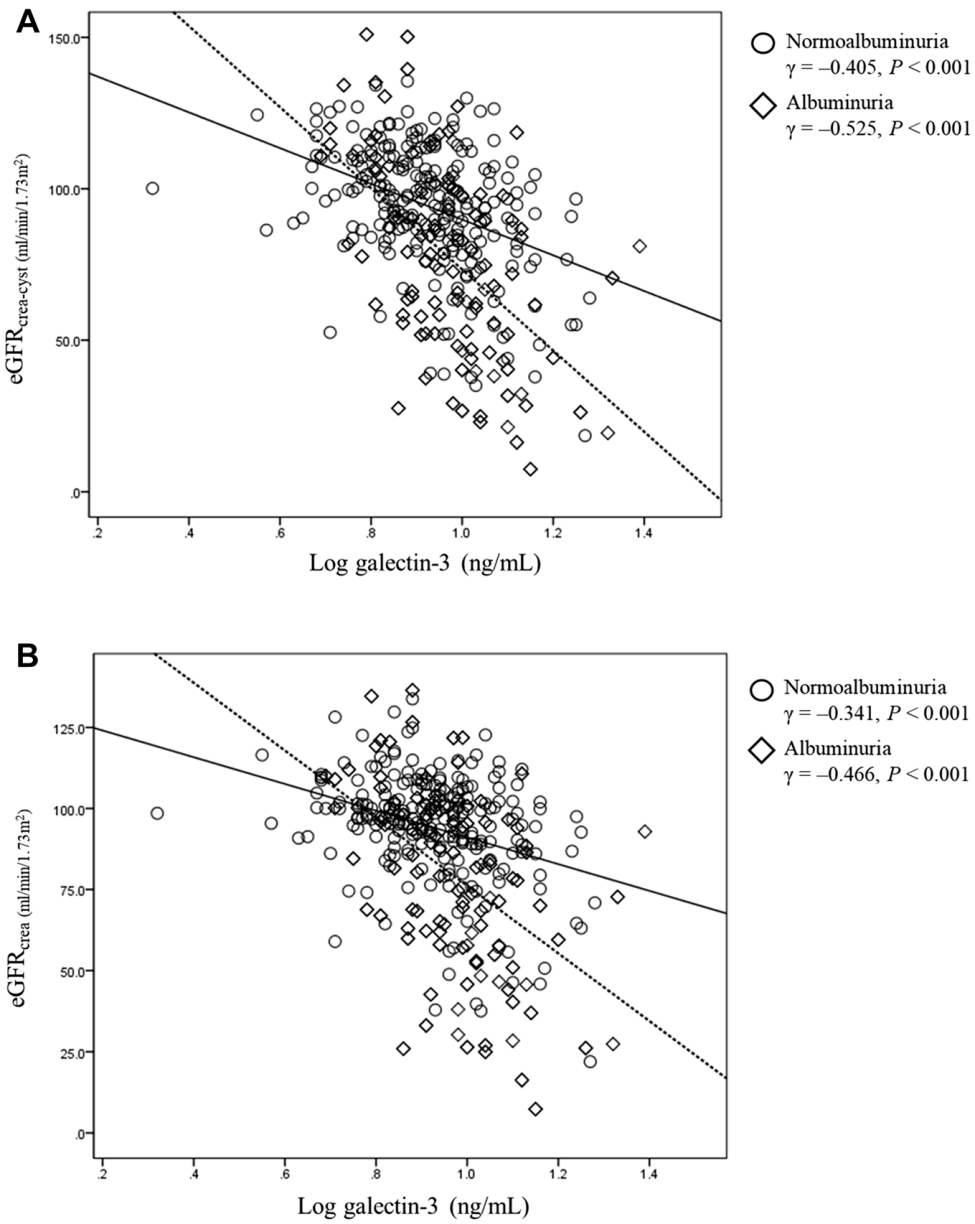
Correlation between galectin-3 concentrations and estimated glomerular filtration rate in patients with T2DM with normoalbuminuria and albuminuria. **A** Correlation between galectin-3 concentrations and eGFR_crea-cyst_. **B** Correlation between galectin-3 concentrations and eGFR_crea_. *eGFR* estimated glomerular filtration rate, *T2DM* type 2 diabetes mellitus.

Pearson’s correlation coefficients between plasma galectin-3 concentrations and eGFR are shown in Fig. 1. Negative correlations were observed between plasma galectin-3 and eGFR_crea-cyst_ in patients with normoalbuminuria (γ = − 0.405, *P* < 0.001) and albuminuria (γ = − 0.525, *P* < 0.001). Likewise, plasma galectin-3 concentrations were negatively correlated with eGFR_crea_ in patients with normoalbuminuria (γ = − 0.341, *P* < 0.001) and albuminuria (γ = − 0.466, *P* < 0.001).

We evaluated the relationship between plasma galectin-3 concentration and eGFR in all patients with T2DM using linear regression models (Table 4). In multiple regression analyses, galectin-3 concentrations were significantly associated with eGFR_crea-cyst_ after adjusting for sex, age, body mass index, smoking, hemoglobin, duration of diabetes, A1C, hyperlipidemia, hypertension, and use of oral hypoglycemic agents (OHAs) and insulin (β = − 0.311, 95% confidence interval [CI] − 74.501 to − 44.854, *P* < 0.001). Furthermore, the significant relationship persisted after adjusting for UACR as a categorical variable (β = − 0.294, 95% CI − 70.804 to − 41.768, *P* < 0.001; Model 3a) and continuous measure (β = − 0.265, 95% CI − 65.192 to − 36.550, *P* < 0.001; Model 3b). Likewise, the results were replicated for eGFR_crea_ in the multiple regression analyses (Table 4). Alternatively, when systolic blood pressure, total cholesterol, and use of ACEi/ARB, statin and sodium-glucose cotransporter 2 inhibitors were included as independent variables in the models and hypertension, hyperlipidemia, and use of OHAs were excluded, plasma galectin-3 concentrations were still associated with eGFR (Supplementary Table 1). Additionally, when patients with use of glucagon-like peptide-1 receptor agonists (n = 2) were excluded in the analyses, almost identical results were found in multiple regression analyses (data not shown).

**Table 4:** Multiple linear regression analyses on eGFR in all patients with T2DM.

Variables		*β*	95% CI	*P*-value	R^2^ (adjusted R^2^)
**eGFR**_**crea-cyst**_					
Galectin-3^†^	Unadjusted	− 0.461	− 106.772, − 70.011	< 0.001	0.212 (0.210)
	Model 1	− 0.356	− 85.270, − 51.420	< 0.001	0.388 (0.382)
	Model 2	− 0.311	− 74.501, − 44.854	< 0.001	0.559 (0.543)
	Model 3a	− 0.294	− 70.804, − 41.768	< 0.001	0.584 (0.567)
	Model 3b	− 0.265	− 65.192, − 36.550	< 0.001	0.606 (0.590)
**eGFR**_**crea**_					
Galectin-3^†^	Unadjusted	− 0.404	− 82.756, − 50.228	< 0.001	0.163 (0.161)
	Model 1	− 0.291	− 62.619, − 33.150	< 0.001	0.370 (0.365)
	Model 2	− 0.248	− 53.883, − 27.760	< 0.001	0.535 (0.518)
	Model 3a	− 0.228	− 50.222, − 24.805	< 0.001	0.566 (0.549)
	Model 3b	− 0.201	− 45.724, − 20.473	< 0.001	0.584 (0.567)

*A1C* glycated hemoglobin, *β* Standardized regression coefficient, *BMI* body mass index, *CI* confidence interval, *eGFR* estimated glomerular filtration rate, *Hgb* hemoglogin, *OHA* oral hypoglycemic agent, *T2DM* type 2 diabetes mellitus, *UACR* urinary albumin-to-creatinine ratio.

^†^Data were logarithmically transformed before analysis.

Model 1: adjusted for sex and age.

Model 2: adjusted for BMI, smoking, duration of diabetes^†^, A1C, Hgb, hyperlipidemia, hypertension, and use of insulin and OHAs, in addition to the variables in Model 1.

Model 3a: adjusted for all confounders in Model 2 plus UACR as albuminuria status (yes/no).

Model 3b: adjusted for all confounders in Model 2 plus UACR as a continuous variable^†^.

## Discussion

In this study, we found that galectin-3 concentration was negatively associated with eGFR in patients with T2DM. Moreover, this association was independent of albuminuria status. Because galectin-3 is related to tissue injury^14,16^, our findings suggest a possible role of galectin-3 as an indicator of early diabetic nephropathy in patients with T2DM.

Galectin-3 has recently emerged as a modulator of several biological processes, including adhesion, proliferation, differentiation, and apoptosis^14^. In addition, galectin-3 levels have been suggested to increase following tissue damage and inflammatory stimuli^15^. Previous investigations on the association between galectin-3 and cardiovascular disease indicated that galectin-3 concentrations in circulation might be a potential biomarker for cardiovascular disease^21^. This speculation has been verified by a number of clinical studies showing that higher circulating concentrations of galectin-3 are associated with an increased risk of heart failure, coronary artery disease, ischemic stroke, and cardiovascular mortality^21–23^.

Recent data have suggested a close relationship between galectin-3 concentration and an increased risk of diabetes^24–26^. Increased circulating concentrations of galectin-3 are found in patients with T2DM compared with those without T2DM^24^. Vora et al.^27^ reported that galectin-3 concentration is positively associated with incident T2DM. In addition, mounting evidence suggests that galectin-3 is involved in the development of kidney disease^19^. In experimental studies, renal galectin-3 expression was found to increase after kidney injury^17^. An increase in circulating galectin-3 concentration has been linked to renal injury in humans^19^. In community-based population studies, elevated galectin-3 concentrations were found to be associated with the development of chronic kidney disease^19,28,29^. In addition, Tan et al.^30^ showed that galectin-3 concentration was associated with a twofold increase in serum creatinine levels and incident macroalbuminuria in a longitudinal study of patients with T2DM. In addition, high concentrations of galectin-3 are associated with worsening albuminuria in patients with T2DM^31^. Our data indicated that galectin-3 concentrations were inversely associated with eGFR in patients with T2DM and albuminuria, in agreement with previous findings^30^. Furthermore, we noticed a close association between galectin-3 concentration and eGFR in patients with T2DM with normoalbuminuria. To date, the relationship between galectin-3 and eGFR in patients with T2DM with normoalbuminuria has not been fully understood. Classically, an initial increase in urinary albumin excretion is presumed to precede a decrease in GFR^9,32^. However, recent studies have suggested that a decrease in GFR may begin earlier^32,33^. Consequently, the findings of this study suggest that increased concentrations of galectin-3 might be implicated in kidney injury in patients with T2DM, independently of albuminuria status.

Galectin-3 concentrations have been suggested to be associated with age^30^. In addition, several studies have reported that galectin-3 concentration is associated with glycemic status in patients with T2DM^30,31^. Similarly, plasma galectin-3 concentrations in the present study were associated with diabetes duration, reflecting the total glycemic control over time^34^. Because these elements are also involved in renal dysfunction^35^, the relationship between galectin-3 concentration and eGFR in the present study might be affected by these factors. However, in multivariable analysis, the association between galectin-3 concentrations and eGFR remained significant after adjusting for these confounders, thus implying that these factors did not significantly influence the relationship between galectin-3 concentrations and eGFR.

Despite a strong association between galectin-3 concentration and renal function loss in diabetes, the exact mechanism underlying the association is controversial. Galectin-3 is a potent activator of fibroblasts and may contribute to renal fibrosis^19^. Renal fibrosis is associated with loss of renal function in diabetes^2^. Pharmacological inhibition of galectin-3 attenuates hypertensive nephropathy in rodent models^36^. A phase II study using a galectin-3 inhibitor suggested an improvement in eGFR in patients with chronic kidney disease^37^. However, a contrasting role for galectin-3 in the kidneys has also been reported^19^. Advanced glycation end products (AGEs) are important contributors to the development of diabetic nephropathy^38^. Galectin-3 has a high binding affinity for AGEs^39^ and has been suggested to participate in the degradation of AGEs^19^. Studies using knockout mouse models have shown that targeted disruption of the galectin-3 gene results in accelerated diabetic glomerulopathy^40^. Therefore, further investigations are needed to establish the exact mechanism by which galectin-3 is involved in the pathogenesis of renal function loss in diabetes.

There are several limitations in our study. First, because of the cross-sectional design, the causal and temporal relationship could not be established in this study. Second, although several potential confounding factors were considered in the regression models, the association between galectin-3 and eGFR could still be affected by unmeasured confounders. Another limitation is that the sample size is relatively small. In spite of these limitations, we believe that our data provide valuable information on the relationship between galectin-3 concentration and eGFR in patients with T2DM with and without albuminuria.

In conclusion, galectin-3 concentrations were negatively associated with eGFR in patients with T2DM, independent of albuminuria status. Further large longitudinal investigations are warranted to confirm that galectin-3 predicts an early progressive decline in renal function in patients with T2DM with and without albuminuria.

## Materials and methods

### Participants

In this cross-sectional study, we consecutively recruited 334 patients with T2DM who visited the diabetes clinic of our hospital. T2DM was diagnosed according to the expert committee’s report on the diagnosis and classification of diabetes mellitus^41^. Patients taking glucocorticoids; those with an inflammatory disorder, infection, coronary artery disease, heart failure, peripheral artery disease, stroke, kidney disease unrelated to diabetes (e.g., intrinsic renal disease [nephritis or nephrotic syndrome], acute renal failure due to use of drugs, contrast agents, septic shock, or postrenal disease), chronic liver disease, malignancy, or end-stage kidney disease; those who had undergone kidney transplantation; and those who were on dialysis were excluded. If the patient had a blood pressure ≥ 140/90 mmHg or was taking antihypertensive agents, the patient was considered to have hypertension. If the patient had total cholesterol levels ≥ 6.5 mmol/L or triglyceride levels ≥ 2.3 mmol/L or was taking lipid-lowering agents, the patient was considered to have hyperlipidemia. The study was approved by the ethics committee of Chonnam National University Hospital, and informed consent was obtained from all participants. The study was conducted in accordance with the Helsinki Declaration-based ethical principles for medical research involving human subjects.

### Measurements

Venous blood samples were collected after overnight fasting. Cystatin C was measured using an assay from Gentian (Moss, Norway), traceable to the international calibrator ERM-DA471/IFCC^42^. Creatinine level was measured using the Jaffe method. Plasma galectin-3 concentrations were determined using a human galectin-3 Quantikine enzyme-linked immunosorbent assay kit (R&D Systems, Minneapolis, MN) (inter-assay coefficient of variation < 7% and intra-assay coefficient of variation < 4.0%) following the manufacturer’s instructions. Glycated hemoglobin (A1C) was assayed using ion-exchange liquid chromatography (Tosoh, Tokyo, Japan). Urinary albumin excretion was determined using the urinary albumin-to-creatinine ratio (UACR). The mean values of urinary albumin excretion were determined from two spot urine samples obtained on two consecutive mornings. Normoalbuminuria was defined as UACR < 30 mg/g, and albuminuria was ≥ 30 mg/g. eGFR was calculated using the Chronic Kidney Disease Epidemiology Collaboration (CKD-EPI) formula combining creatinine-cystatin C (eGFR_crea-cyst_)^43^. As an alternative, eGFR was also estimated using the CKD-EPI creatinine formula (eGFR_crea_)^43^.

### Statistical analyses

The sample size was estimated using G*Power 3.1.9.2^44^. Using a two-tailed test, the sample size was determined to detect a medium effect size (correlation coefficient) of 0.3 with α of 0.05 and a power of 0.80. The minimum sample size was estimated to be 84.

Quantitative variables are presented as the mean ± standard deviation (SD) or median (25th, 75th percentile). Qualitative variables are presented as frequencies (percentages). Differences between groups were assessed using Student's t-test and Mann–Whitney U test for parametric and nonparametric continuous variables, respectively. Chi-square test or Fisher’s exact test was used to compare categorical variables. Normal distribution was assessed using both visual inspection of histograms and the Kolmogorov–Smirnov test. Logarithmic transformation for variables with skewed distributions was conducted prior to correlation and regression analyses. Pearson’s correlation analysis was used to assess the association between plasma galectin-3 levels and eGFR. We evaluated the association between galectin-3 concentration and eGFR using a multiple linear regression model. Variables were used as covariates in the regression model if they were previously shown to relate to the risk of renal function decline^2,45^ and/or exhibited significant differences between patients according to median galectin-3 in either normoalbuminuria or albuminuria group. These covariates are as follows: age, body mass index, smoking, duration of diabetes, A1C, hemoglobin, hyperlipidemia, hypertension, and anti-diabetic therapy. Sex was also considered as a covariate. We adjusted for sex and age (Model 1). Model 2 included adjustments for body mass index, smoking, duration of diabetes, A1C, hemoglobin, hyperlipidemia, hypertension, and use of insulin and oral hypoglycemic agents in addition to the parameters in Model 1. In Model 3a, we adjusted for all parameters in Model 2 plus UACR as albuminuria status (yes/no). In Model 3b, we adjusted for all parameters in Model 2 plus UACR as the continuous variable. Multicollinearity was examined using the variance inflation factor. A variance inflation factor > 10 was excluded from the models. All analyses were conducted using the SPSS software version 20.0 (SPSS, Chicago, IL, USA). *P* significance was set at < 0.05.

## Supplementary Information

Supplementary Information.

## Data Availability

The datasets used and/or analyzed during the current study available from the corresponding author on reasonable request.
