# Supplementary material for: Plasma galectin-3 concentration and estimated glomerular filtration rate in patients with type 2 diabetes with and without albuminuria
Source: Sci Rep. 2022 Sep 29;12:16328. doi: 10.1038/s41598-022-20860-x (PMC9522850; doi:10.1038/s41598-022-20860-x)
Supplement: Supplementary file 1 — Supplementary Information. [file 41598_2022_20860_MOESM1_ESM.doc]

**Plasma galectin-3 concentration and estimated glomerular filtration rate in patients with type 2 diabetes with and without albuminuria**

Jin Ook Chung^1*^, Seon-Young Park^2^, Seung Baek Lee^3,4^, Na-Ri Kang^1^, Dong Hyeok Cho^1^, Dong Jin Chung^1^, Min Young Chung^1^

^1^Division of Endocrinology and Metabolism, Department of Internal Medicine, Chonnam National University Medical School, Gwangju, Republic of Korea

^2^Division of Gastroenterology and Hepatology, Department of Internal Medicine,

Chonnam National University Medical School, Gwangju, Republic of Korea

^3^Division of Radiology, Mayo Clinic, Rochester, MN, USA

^4^Department of Molecular Pharmacology and Experimental Therapeutics, Mayo Clinic, Rochester, MN, USA

Supplementary Table 1. Multiple linear regression analyses on eGFR in all patients with T2DM when systolic BP, total cholesterol, and use of ACEi/ARB, statin and SGLT2i were included independent variables and hypertension, hyperlipidemia, and OHAs were excluded

| Variables |  | *β* | 95% CI | *P*-value | R^2^ (adjusted R^2^) |
| --- | --- | --- | --- | --- | --- |
| eGFR_crea-cyst_ |  |  |  |  |  |
| Galectin-3^†^ | Unadjusted | -0.461 | -106.772, -70.011 | <0.001 | 0.212 (0.210) |
|  | Model 1 | -0.356 | -85.270, -51.420 | <0.001 | 0.388 (0.382) |
|  | Model 2 | -0.325 | -76.765, -47.399 | <0.001 | 0.573 (0.554) |
|  | Model 3a | -0.310 | -73.613, -44.696 | <0.001 | 0.591 (0.572) |
|  | Model 3b | -0.282 | -67.979, -39.539 | <0.001 | 0.615 (0.597) |
| eGFR_crea_ |  |  |  |  |  |
| Galectin-3^†^ | Unadjusted | -0.404 | -82.756, -50.228 | <0.001 | 0.163 (0.161) |
|  | Model 1 | -0.291 | -62.619, -33.150 | <0.001 | 0.370 (0.365) |
|  | Model 2 | -0.261 | -55.595, -29.749 | <0.001 | 0.549 (0.525) |
|  | Model 3a | -0.243 | -52.454, -27.146 | <0.001 | 0.574 (0.553) |
|  | Model 3b | -0.217 | -48.003, -22.928 | <0.001 | 0.593 (0.573) |

^†^Data were logarithmically transformed before analysis.

A1C, glycated hemoglobin; ACEi, angiotensin converting enzyme inhibitor; ARB, angiotensin II receptor blocker; *β*, Standardized regression coefficient; BMI, body mass index; BP, blood pressure; CI, confidence interval; eGFR, estimated glomerular filtration rate; Hgb, hemoglogin; SGLT2i, sodium-glucose cotransporter 2 inhibitor; T2DM, type 2 diabetes mellitus; UACR, urinary albumin-to-creatinine ratio

Model 1: adjusted for sex and age.

Model 2: adjusted for BMI, smoking, duration of diabetes^†^, A1C, Hgb, systolic BP, total cholesterol^†^, and use of ACEi/ARB, statin, insulin and SGLT2i, in addition to the variables in Model 1.

Model 3a: adjusted for all confounders in Model 2 plus UACR as albuminuria status (yes/no).

Model 3b: adjusted for all confounders in Model 2 plus UACR as a continuous variable^†^
